# Mandibular and dental measurements for sex determination using machine learning

**DOI:** 10.1038/s41598-024-59556-9

**Published:** 2024-04-26

**Authors:** Erika Calvano Küchler, Christian Kirschneck, Guido Artemio Marañón-Vásquez, Ângela Graciela Deliga Schroder, Flares Baratto-Filho, Fábio Lourenço Romano, Maria Bernadete Sasso Stuani, Mírian Aiko Nakane Matsumoto, Cristiano Miranda de Araujo

**Affiliations:** 1 Department of Orthodontics, Medical Faculty, University Hospital Bonn, Welschnonnenstr. 17, 53111 Bonn, Germany; 2 Department of Pediatric Dentistry, School of Dentistry of Ribeirão Preto, University of São Paulo, Av. do Café s/n, Ribeirão Preto, São Paulo 14040-904 Brazil; 3 Postgraduate Program in Communication Disorders, Tuiuti University of Paraná, R. Padre Ladislau Kula 395, Curitiba, Paraná 82010-210 Brazil; 4 School of Dentistry, Tuiuti University of Paraná, R. Padre Ladislau Kula 395, Curitiba, Paraná 82010-210 Brazil; 5 Department of Dentistry, University of the Region of Joinville (Univille), R. Paulo Malschitzki 10, Joinville, Santa Catarina 89219-710 Brazil

**Keywords:** Sex determination, Artificial intelligence, Deep learning, Oral anatomy, Machine learning

## Abstract

The present study tested the combination of mandibular and dental dimensions for sex determination using machine learning. Lateral cephalograms and dental casts were used to obtain mandibular and mesio-distal permanent teeth dimensions, respectively. Univariate statistics was used for variables selection for the supervised machine learning model (alpha = 0.05). The following algorithms were trained: logistic regression, gradient boosting classifier, k-nearest neighbors, support vector machine, multilayer perceptron classifier, decision tree, and random forest classifier. A threefold cross-validation approach was adopted to validate each model. The areas under the curve (AUC) were computed, and ROC curves were constructed. Three mandibular-related measurements and eight dental size-related dimensions were used to train the machine learning models using data from 108 individuals. The mandibular ramus height and the lower first molar mesio-distal size exhibited the greatest predictive capability in most of the evaluated models. The accuracy of the models varied from 0.64 to 0.74 in the cross-validation stage, and from 0.58 to 0.79 when testing the data. The logistic regression model exhibited the highest performance (AUC = 0.84). Despite the limitations of this study, the results seem to show that the integration of mandibular and dental dimensions for sex prediction would be a promising approach, emphasizing the potential of machine learning techniques as valuable tools for this purpose.

## Introduction

Human sexual dimorphism is a widely studied field and explores many psychological and biological characteristics. Although the face is a well-known biological billboard of human identity and it is the dimorphic trait most extensively investigated^1^, humans also exhibit significant sexual dimorphism in other traits of the craniofacial complex. Several studies in different populations attempted to identify the distinction between sexes by evaluating craniofacial structures^2–5^, such as teeth dimensions^6–8^ and mandible size and characteristics^9–11^.

Mandible is considered in the literature as one of the strongest craniofacial bones for gender identification^11^. Its relatively indestructible and morphological variation contain safe parts to be used in sex determination. A previous systematic review evaluated several mandibular parameters explored for sex dimorphism, showing that some mandibular measurements present sexual dimorphism^9^.

Teeth are well-known as the most indestructible structure of the human body and are vital key evidence in several investigations. Teeth are preserved in the closed cavities of the mouth and are generally resistant to environmental threats^12^. Morphological and, especially, metric parameters of permanent teeth also present sexual dimorphism^13–18^. Permanent teeth dimensions, such as the mesio-distal size, are the most frequently assessed odontometric variables for sex determination^8,13^. Males have larger teeth crowns than females in contemporary human populations, however this dimorphism varies depending on the population^19^.

In the past years, data science techniques, such as machine learning, have been used for sex determination^20–22^. Machine learning is a subset of artificial intelligence that has the capability to make predictions without being explicitly programmed to do, using mathematical models generated from a sample, which is a ‘training’ data^23^. Some studies used machine learning to explore craniofacial structures (including mandibular parameters and teeth dimension) for sex determination^7,20,21,24,25^. These previous studies demonstrated that mandibular measurements and dental size are parameters suitable for sex determination, presenting a good overall accuracy of their models^7,20,21,24,25^, however none of them evaluated teeth and craniofacial measurements in the same study. The combination of mandibular measurements and dental size in the same model could increase the accuracy of the model. Therefore, the present study aimed to test the integration of mandibular and dental dimensions to improve sex determination using machine learning.

## Results

A total of 108 individuals were included in the study (51% females and 49% males); age ranging from 9 to 40 years old (15.7 ± 7.9 years).

The univariate analysis showed that two variables (Go–Pg, mandibular body length; and SNB) were not significantly different between males and females (*p* > 0.05); therefore, these were not integrated into the prediction model. The mean values of the mandibular and dental measurements evaluated are available in Table 1.

**Table 1:** Mandibular and dental measurements according to the sex.

Measurement	n	Mean (SD)	*P* value	Power test
Mandibular Length (Co–Gn)				
Male	53	118.63 (13.18)	0.006	0.78
Female	55	112.72 (8.18)		
Mandibular body length (Go–Pg)—mm				
Male	53	69.20 (8.84)	0.121	0.30
Female	55	66.84 (6.77)		
Mandibular ramus height (Co–Go)—mm				
Male	53	60.75 (8.71)	< 0.001	0.96
Female	55	55.40 (5.62)		
SNB				
Male	53	81.41 (5.69)	0.052	0.50
Female	55	79.53 (4.08)		
Mandibular divergence—Y-axis (S.Gn–SN)				
Male	53	58.6 (4.8)	0.015	0.70
Female	55	60.9 (4.7)		
Upper incisors				
Male	53	8.07 (0.49)	< 0.001	0.95
Female	55	7.69 (0.58)		
Upper canines				
Male	53	7.91 (0.50)	0.004	0.85
Female	55	7.59 (0.60)		
Upper premolars				
Male	55	7.08 (0.51)	0.011	0.74
Female	53	6.82 (0.52)		
Upper first molars				
Male	53	9.99 (0.53)	< 0.001	0.98
Female	53	9.57 (0.51)		
Lower incisors				
Male	52	5.91 (0.38)	0.009	0.75
Female	54	5.71 (0.39)		
Lower canines				
Male	52	7.14 (0.49)	< 0.001	0.99
Female	54	6.59 (0.44)		
Lower premolars				
Male	51	7.39 (0.69)	0.031	0.61
Female	54	7.10 (0.63)		
Lower first molar				
Male	50	11.29 (0.69)	< 0.001	0.99
Female	53	10.68 (0.58)		

**Figure 1:**
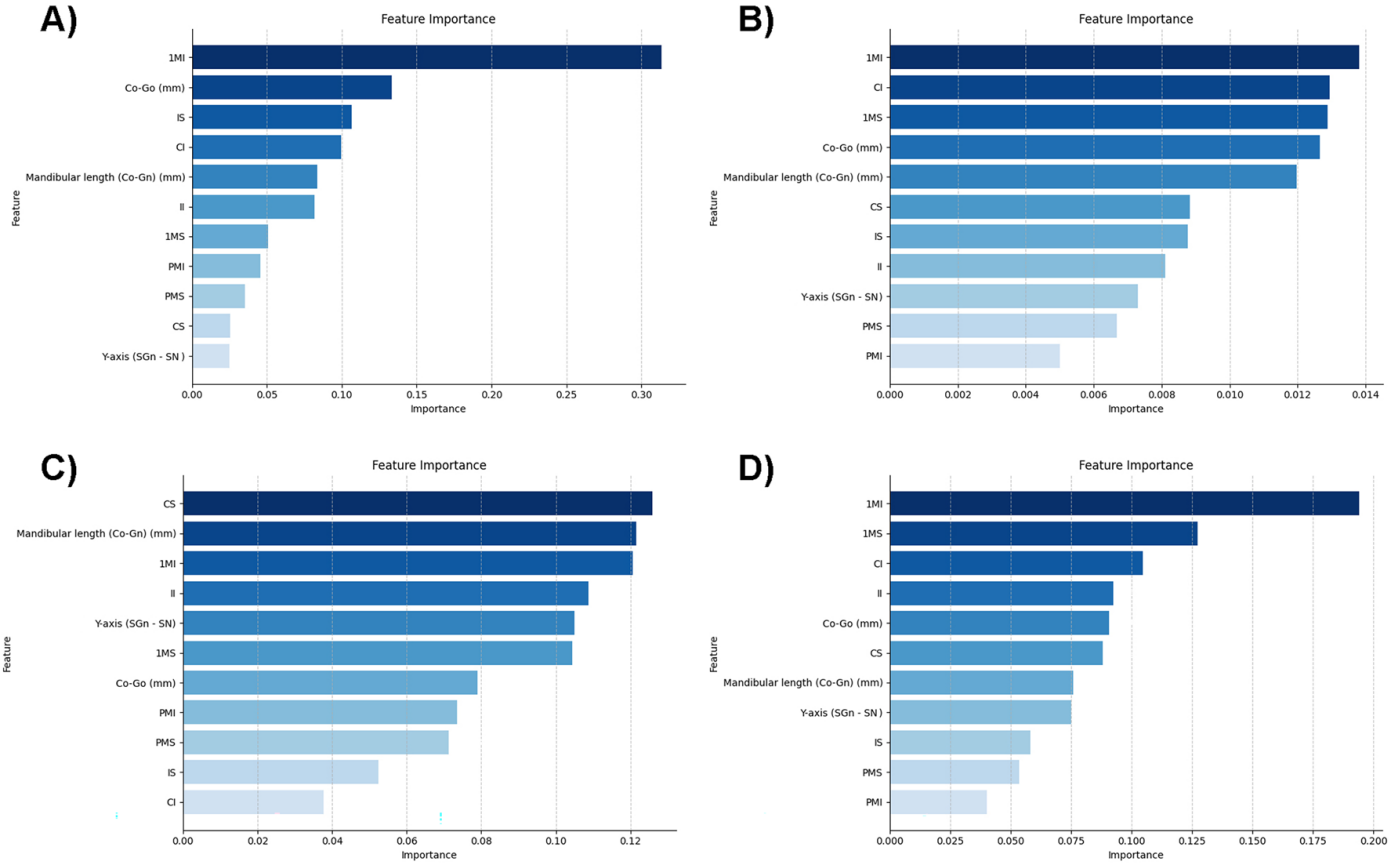
Results of feature importance analysis from four machine learning models. (**A**) Gradient Boosting Classifier, (**B**) Logistic Regression, (**C**) Decision Tree, (**D**) Random Forest Classifier.

Three mandibular-related measurements and eight dental size-related dimensions were used to train the machine learning models. Among the dental size-related variables, the mesio-distal size of the lower first molar demonstrated higher relevance in three out of the four evaluated models (Fig. 1). The mandibular ramus height (Co–Go) exhibited the greatest predictive capability in three out of the four analyzed models among mandibular-related variables (Fig. 1). The performances of the tested predictive models, along with the hyperparameters considered optimal for each model, are detailed in Table 2.

**Table 2:** Summary of metrics obtained for the cross-validation and test stages of the models, along with their respective optimal hyperparameters.

Model	Optimal hyperparameters (random_state = 42)	Cross-validation results (cv = 3)	Test data results
Logistic regression	C: 0.001	Accuracy = 0.74	Accuracy = 0.79
	max_iter: 50	Precision = 0.74	Precision = 0.82
	penalty: l2	Recall = 0.74	Recall = 0.79
	l1_ratio: 0.2	F1-Score = 0.74	F1-Score = 0.79
	solver: liblinear		
Gradient boosting classifier	n_estimators: 2000	Accuracy = 0.67	Accuracy = 0.71
	learning_rate: 0.1	Precision = 0.67	Precision = 0.74
	criterion: friedman_mse	Recall = 0.67	Recall = 0.71
	max_depth: 3	F1-Score = 0.67	F1-Score = 0.71
	loss: deviance		
K-nearest neighbors	n_neighbors: 10	Accuracy = 0.71	Accuracy = 0.75
	weights: distance	Precision = 0.73	Precision = 0.85
	leaf_size: 1	Recall = 0.71	Recall = 0.75
	p: 10	F1-Score = 0.70	F1-Score = 0.75
Support vector machine	kernel: linear	Accuracy = 0.70	Accuracy = 0.75
	C: 0.1	Precision = 0.71	Precision = 0.85
	gamma: auto	Recall = 0.70	Recall = 0.75
		F1-Score = 0.68	F1-Score = 0.75
MLPClassifier	activation: tahn	Accuracy = 0.69	Accuracy = 0.66
	alpha: 0.01	Precision = 0.68	Precision = 0.71
	hidden_layer_sizes: 10	Recall = 0.69	Recall = 0.67
	learning_rate_init: 0.01	F1-Score = 0.68	F1-Score = 0.67
	max_iter: 100		
	solver: lbfgs		
Decision tree	criterion: entropy	Accuracy = 0.64	Accuracy = 0.58
	max_depth: none	Precision = 0.64	Precision = 0.63
	splitter: random	Recall = 0.64	Recall = 0.58
		F1-Score = 0.64	F1-Score = 0.59
Random forest classifier	max_depth: none 10	Accuracy = 0.73	Accuracy = 0.62
	n_estimators: 50	Precision = 0.73	Precision = 0.66
	min_samples_split: 2	Recall = 0.73	Recall = 0.62
	min_samples_leaf: 4	F1-Score = 0.73	F1-Score = 0.63
	criterion: entropy		
	max_features: auto		

**Figure 2:**
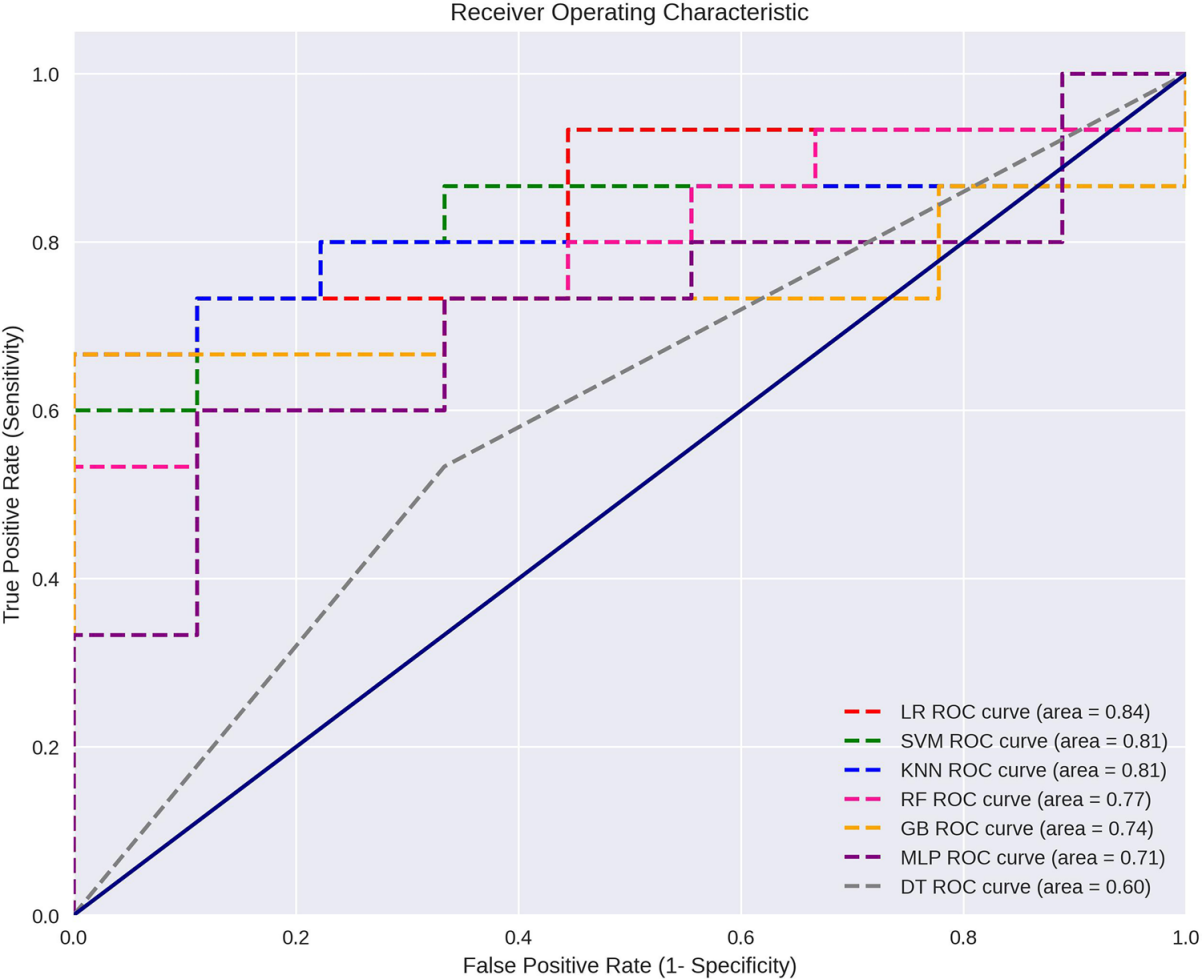
Evaluation of Classification Models using ROC Curves. *LR* Logistic Regression, *SVM* Support Vector Machine, *KNN* K-Nearest Neighbors, *GB* Gradient Boosting, *MLP* Multilayer Perceptron, *RF* Random Forest, *DT* Decision Tree.

Analysis of the models' accuracy revealed a variation ranging from 0.64 to 0.74 during the cross-validation stage, while for the test data, this variation ranged from 0.58 to 0.79. The logistic regression model exhibited the highest average performance, with an area under the curve (AUC) of 0.84 (Fig. 2).

## Discussion

Some methods that explore sexual dimorphism are based on the structures belonging to the craniofacial complex, including mandibular^9^ and teeth measurements^14^. It is well known in the literature that the craniofacial complex exhibits significant sexual dimorphism^26,27^ and that these traits can facilitate accurate sex determination. Although the use of craniofacial landmarks^8–11,13^ and measurements from orthodontic records^7^ have been used for sexual discrimination for decades, it is important to emphasize that our study brings new models once combined mandibular measurements, dental measurements, and artificial intelligence to explore this issue.

Sex determination is essential in various disciplines, including anthropology and forensic. In forensic it is a primary task when dealing with human skeletal remains. However, the understanding of the phenotypes that present sexual dimorphism in humans also brings some clues in the etiological mechanisms involved in these traits. Characteristics with a remarkable sexual dimorphism are phenotypic expression of chromosomal, gonadal, and hormonal level. It is well known that sex chromosomes are involved in dental tissues formation^28,29^. Studies with different designs concluded that tooth development is, in part, controlled by sex-related genes. Consequently, structures of human permanent dentition exhibit sex differences. Previous studies support that the maxillary and mandibular canine show the largest dimension variation of sexual dimorphism^8,13^. In our study, although mesio-distal size of the canines presented a strong statistical difference among sexes, the lower first molar exhibited greater predictive capability, demonstrating higher relevance in three out of the four evaluated models.

One important limitation that should be emphasized in our study is that different from dental measurements, in which mesio-distal sizes do not change according to the age, mandibular measurements vary according to the age. Sexual dimorphism reaches full expression after puberty, due to the influence of androgens and estrogens^30^. The sample used here to create the model included mainly teenagers and adults. Although some variability in the mandible size according to the age might exist, this is reduced due the fact that young children were not included. The age range of our sample has a significant role in the generalizability and applicability of the developed model. Although the age variation could reduce the accuracy of the model due to the complexity added to the identification of the patterns of different ages, it is important to highlight that this inclusion reflects the reality of the target population, specially in the forensic practice. Therefore, although the age range might have impacted the model’s accuracy, it increased the external validity and reflects its capability in different unknown environments.

The determination of sex and identification of population affinity are two important aspects of forensic investigation. In our study, an orthodontic population from a southeast region of Brazil was investigated. Different from the pelvic bone, the main disadvantage of the skull is that sexual dimorphism of the craniofacial complex structures is population specific^31^. Therefore, it is important to emphasize that this is a preliminary study that focused only on a specific Brazilian sample and that this study should be replicated in different populations. The fact that an orthodontic sample has been used should also be highlighted. Although other previous studies also used orthodontic sample to investigate sex discrimination^7^, conventional two-dimensional lateral cephalometric analysis present limitation in finding accurate measurement point due to overlapping of some bony structures.

Several previous studies extensively studied permanent human dentition to estimate sex^8^ with inconsistent findings^6^. Therefore, in our study mandibular measurements were added to increase the estimation accuracy level. Like this study, previous results investigated the sexual dimorphism of some parameters such as mandibular ramus length, ramus width, and gonial angle^11^. In a previous study^32^ the mandibular ramus, presented a large difference among sexes. Another research^33^ tried to determine sex using the mandible and they concluded that although different tendencies exist between the mandible of males and females, the extent of these differences is not enough to predict the sex of a single individual.

It is also important to mention that models to evaluate sex, covers many metric and non-metric parameters. However, in our study only metric parameters were included to avoid subjectivity. An important aspect to emphasize is the use of machine learning techniques to enhance the accuracy of our analyses. Machine learning is a subset of artificial intelligence that relies on algorithms to predict outcomes based on datasets. The primary goal of machine learning is to enable machines to learn from data and solve problems without human intervention^7,20^. Previous studies evaluated craniofacial traits to estimate sex using artificial intelligence. Toy et al.^20^ and Toneva et al.^21^ investigated computerized tomography (CBCT) images of the cranium and used parameters of the whole skull. Baban et al.^24^, also used CBCT to test the accuracy of the sex identification based on linear and volumetric measurements of the mandible. Senol et al.^25^ evaluated canines and molars measurements using CBCT for sex determination, while Anic-Milosevic et al.^7^ used dental cast from orthodontic records and used dental measurements for sex determination. Although their data showed a good accuracy, none of these previous studies added dental and craniofacial measurements in the same model. To the best of our knowledge, our study was the first to include bone and teeth measurements.

In our study, current estimates reveal a good overall accuracy of the model, especially for the logistic regression model. However, when considering metrics beyond AUC, it is observed that the precision values of this model were lower compared to the KNN and SVM models. It is also noteworthy that all metric values showed a decrease in cross-validation results. These findings align with the precision of previous studies^25,34,35^, which employed larger samples combined with Machine Learning and Deep Learning techniques. This suggests a promising outlook for the model built in this study. One important aspect to be highlighted is the age heterogeneity of the sample. Although this heterogeneity can impact the model accuracy because mandibular size ranges according to the age, this sample variability reflects the forensic reality, in which remains of subjects of different ages are analysed. The sample size is one of the limitations of the present study; however, the variables included in the model showed adequate statistical power and demonstrated statistical significance in the univariate analysis.

It is plausible to hypothesize that a more precise model could be achieved with a more homogeneous sample and a larger sample size. Briefly, the findings and the design of this study may contribute to the knowledge of different fields, such as anthropology, forensic science, orthodontics, and craniofacial biology, providing valuable insights for research and practical applications.

## Methods

This cross-sectional study evaluated orthodontic records from patients in treatment at the School of Dentistry of Ribeirão Preto, University of São Paulo. This study was conducted in accordance with the Declaration of Helsinki and approved by the Human Ethics Committee of the School of Dentistry of Ribeirão Preto, University of São Paulo, São Paulo, Brazil (3.150.551). Informed consent was obtained from all patients/children and/or their parents/legal guardians (in the case of minors).

The studied samples are orthodontic Brazilian patients from Ribeirão Preto, a city with an estimated population of 720,216 inhabitants in 2010, located in São Paulo state. Ribeirão Preto is a city with an admixed population, in which they self-report their ethnicity as: 69.8% European ancestry (mainly Portuguese and Italian ancestry), 6.4% African ancestry (mainly west central Africa), 0.9% Asian ancestry (mainly East Asia and Middle Eastern), 0.1% Indigenous Peoples, and 22.8% mixed^36^.

Lateral cephalograms and dental casts of the maxilla and mandible were used for analyses. Records from individuals with underlying syndromes or congenital alterations were not included in this study.

### Study variables and data collection

Tracings from lateral cephalograms were conducted by a proficient and calibrated orthodontist as previously described^37^. The following linear and angular mandibular measurements were evaluated: mandibular total length (Co–Gn), mandibular body length (Go–Pg), mandibular ramus height (Co–Go), Steiner’s SNB angle, and the Y-axis (S.Gn–SN).

Dental casts from maxilla and mandible were used to measure the maximum crown dimensions of permanent teeth in the mesio-distal direction. Only fully erupted teeth, without proximal dental caries or restoration, or significant crown abnormality were evaluated. Teeth mesio-distal size was defined as the maximum distance between the mesial and distal anatomical proximal contact points of the tooth on a line perpendicular to the long axis of the tooth crown. Second and third molars were not included. Only one previously calibrated operator measured all teeth. Each tooth was measured twice, and the arithmetic means were calculated for further analyses. If measurements differed by more than 0.2 mm, the measurements were repeated as previously described^15,16^.

An adequate intra-examiner reproducibility was observed for the skeletal and dental measurements performed^15,16,38^.

### Data analysis and model construction

For selection of the variables to be included in the supervised machine learning model, a univariate analysis using the Student's t test for independent samples was initially performed (α = 0.05). The power of the test obtained for each comparison was calculated using the statistical software GPower version 3.1.9.6.

Prior to model construction, data preprocessing and cleaning were performed. Outliers were identified to provide a deeper understanding of the dataset. These steps were undertaken to prepare the data for subsequent analysis. In order to reduce the dimensionality of the data and mitigate the influence of multicollinearity on predictive models (due to the similarity between dental groups and their respective contralateral groups), the average dental size was calculated for each dental group. Thus, teeth from the same arch, belonging to the same dental group, were aggregated into a single input variable for the analysis.

**Figure 3:**
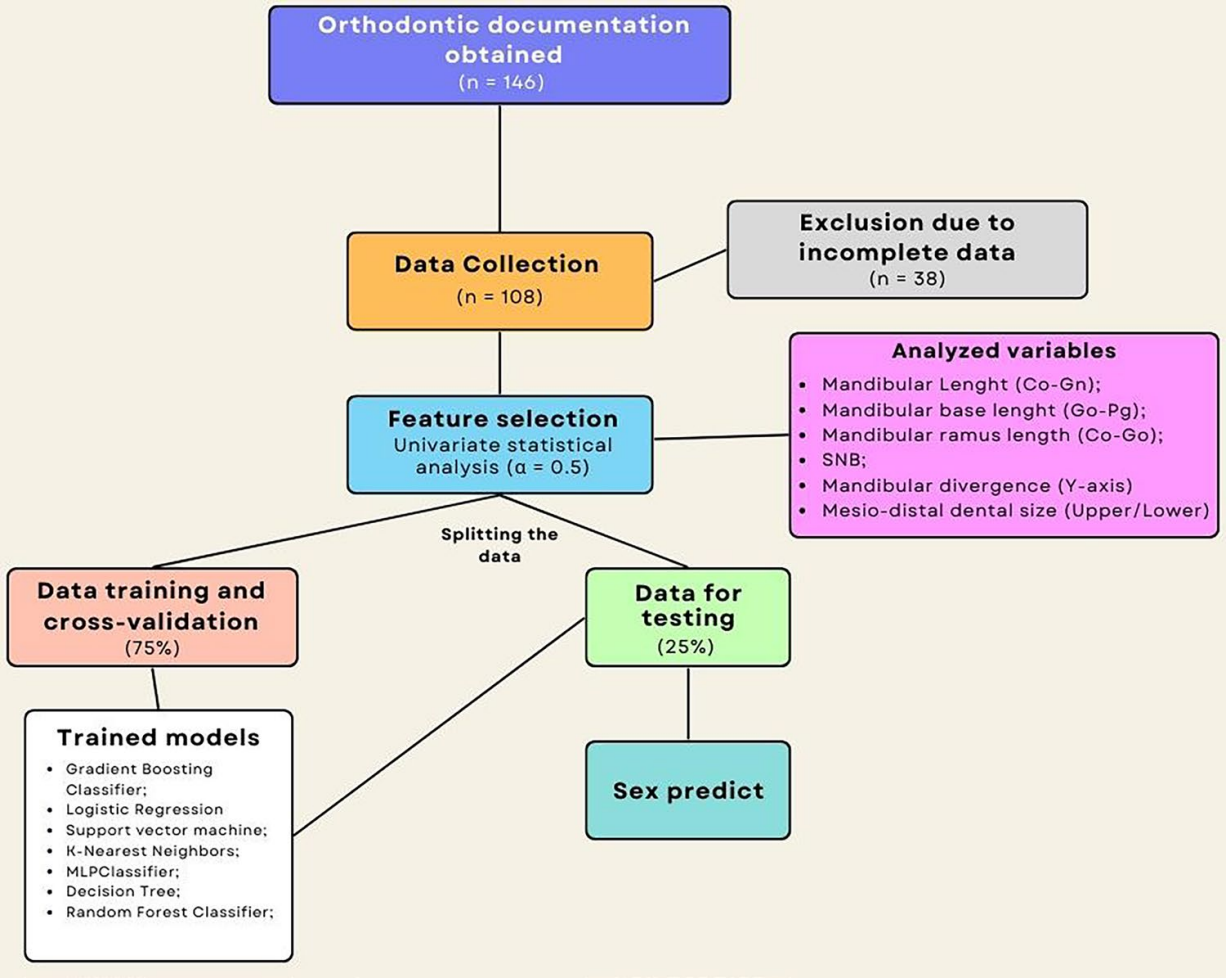
Flowchart diagram illustrating the data analysis process using machine learning models.

For the construction of predictive models, the following supervised machine learning algorithms were trained: Logistic Regression, Gradient Boosting Classifier, K-Nearest Neighbors (KNN), Support Vector Machine (SVM), Multilayer Perceptron Classifier (MLP), Decision Tree, and Random Forest Classifier (Fig. 3). For each model, the Grid Search method was employed, entailing the systematic evaluation of predefined hyperparameter combinations, thus facilitating the identification of the optimal configuration for each model.

### Training, cross-validation, and test

To build predictive models, 75% of the dataset was allocated for both model training and cross-validation implementation. The remaining 25% was set aside for evaluating the predictive capacity of each model. The data was split into training and testing sets using the 'train_test_split' function from the 'sklearn.model_selection' library. The performance assessment, using the k-fold cross-validation technique, involved splitting the data into k subsets, with the model being trained k times. In each iteration, k-1 subsets were used for training, and the remaining subset was used for validation. This approach facilitated the calculation of the average cross-validation results, resulting in a more reliable estimate of the model's performance concerning unseen data. In this study, a threefold cross-validation approach was adopted to validate each model.

Additionally, for each predictive model, the AUC were computed, and ROC curves were constructed. This involved calculating the false positive rate (FPR) and true positive rate (TPR), as well as the area under the ROC curve (AUC). Metrics such as accuracy, recall, precision, and F1 Score were calculated for each model. Furthermore, the feature importance evaluation function from the Scikit-learn library was employed to visually identify the most relevant variables in each model's formulation. This step is important for understanding which features have a greater influence on the model's predictive ability. However, this evaluation was not conducted for the KNN, SVM, and MLP models due to the specificities of these algorithms, which do not operate with this function. The entire analytical process was conducted using the Python programming language (Supplementary Data 1) within the Google Colab environment.

ROC curves were plotted for the different predictive models using the 'matplotlib.pyplot' library. Each curve represents the trade-off between the true positive rate (sensitivity) and the false positive rate (1-specificity) across different threshold values. The area under the ROC curve (AUC) was calculated for each model, providing a measure of its overall performance in binary classification tasks.

### Supplementary Information

Supplementary Information.

## Data Availability

The data that support the findings of this study are available from the corresponding author upon reasonable request.
