# Supplementary material for: Mandibular and dental measurements for sex determination using machine learning
Source: Sci Rep. 2024 Apr 26;14:9587. doi: 10.1038/s41598-024-59556-9 (PMC11053013; doi:10.1038/s41598-024-59556-9)
Supplement: Supplementary file 1 — Supplementary Information. [file 41598_2024_59556_MOESM1_ESM.pdf]

Library and packages

```
!pip install -U mlflow --quiet
import os
import pandas as pd
import numpy as np
from sklearn.model_selection import train_test_split
from sklearn.preprocessing import StandardScaler
import warnings
import matplotlib.pyplot as plt
from matplotlib.colors import ListedColormap
from sklearn.svm import SVC
from sklearn.metrics import accuracy_score
from sklearn.neighbors import KNeighborsClassifier
from sklearn.linear_model import LogisticRegression
from sklearn.preprocessing import StandardScaler

warnings.filterwarnings('ignore')
```

Dataset

```
data = pd.read_excel('/content/cefalometriasexo.xlsx')
```

```
data.rename(columns={'Gender Male:1, Female:2': 'Sex'}, inplace=True)
mapeamento_sex = {1: 'Masculino', 2: 'Feminino'}
data['Sex'] = data['Sex'].replace(mapeamento_sex)
```

Data preprocessing

```
first_column = data.pop('Sex')
data.insert(0, 'Sex', first_column)
data.loc[0, 'Dente 45'] = 10.94
colunasfinal =['Sex', 'Age',
       '\xa0\xa0\xa0Length of Mand Base (Go-Pg)(mm)', '\xa0Co-Go (mm)',
       'SNB', 'Mandibular length (Co-Gn) (mm)',
       'Y-axis (SGn - SN -7)','Dente 11', 'Dente 12', 'Dente 13', 'Dente 14', ' Dente 15',
       'Dente 16', 'Dente 21', 'Dente 22', ' Dente 23', 'Dente 24',
       'Dente 25', 'Dente 26', 'Dente 31', 'Dente 32', 'Dente 33',
       'Dente 34', 'Dente 35', 'Dente 36', 'Dente 41', 'Dente 42',
       'Dente 43', 'Dente 44', 'Dente 45', 'Dente 46']

data = data[colunasfinal].copy()

#gerar coluna total_inf
data['total_inf'] = data[['Dente 31', 'Dente 32', 'Dente 34', 'Dente 35',
       'Dente 36', 'Dente 41', 'Dente 42', 'Dente 44', 'Dente 45', 'Dente 46',
       'Dente 33', 'Dente 43']].sum(axis=1)

#gerar coluna total_sup
data['total_sup'] = data[['Dente 11', 'Dente 12', 'Dente 14', ' Dente 15',
       'Dente 16', 'Dente 21', 'Dente 22', 'Dente 24', 'Dente 25', 'Dente 26',
       'Dente 13', ' Dente 23']].sum(axis=1)

# Calculando a média entre as colunas e criando a nova coluna '1PMS'
data['IS'] = data[['Dente 11', 'Dente 21', 'Dente 12', 'Dente 22']].mean(axis=1)
data['CS'] = data[['Dente 13', ' Dente 23']].mean(axis=1)
data['PMS'] = data[['Dente 14', 'Dente 24',' Dente 15', 'Dente 25']].mean(axis=1)
data['1MS'] = data[['Dente 16', 'Dente 26']].mean(axis=1)
data['II'] = data[['Dente 31', 'Dente 41','Dente 32', 'Dente 42']].mean(axis=1)
data['CI'] = data[['Dente 33', 'Dente 43']].mean(axis=1)
data['PMI'] = data[['Dente 34', 'Dente 44','Dente 35', 'Dente 45']].mean(axis=1)
data['1MI'] = data[['Dente 36', 'Dente 46']].mean(axis=1)
```

```
##Verificação de Outliers
Q1 = data['total_inf'].quantile(0.25)
Q3 = data['total_inf'].quantile(0.75)
IQR = Q3 - Q1
lower_bound = Q1 - 1.5 * IQR
upper_bound = Q3 + 1.5 * IQR
outliers = data[(data['total_inf'] < lower_bound) | (data['total_inf'] > upper_bound)]
outliers
```

```
##Identificando Outliers sup
Q1 = data['total_sup'].quantile(0.25)
Q3 = data['total_sup'].quantile(0.75)
IQR = Q3 - Q1
lower_bound = Q1 - 1.5 * IQR
upper_bound = Q3 + 1.5 * IQR
outliers = data[(data['total_sup'] < lower_bound) | (data['total_sup'] > upper_bound)]
linhas_outliers = outliers.any(axis=1)
print("Linhas com outliers:")
print(linhas_outliers[linhas_outliers].index.tolist())

outliers
```

```
colunasfinal =['Sex',
       '\xa0Co-Go (mm)','Mandibular length (Co-Gn) (mm)',
              'Y-axis (SGn - SN -7)', 'II','CI', 'PMI','1MI','IS', 'CS', 'PMS', '1MS']
data = data[colunasfinal].copy()
data.dropna(inplace=True)
```

Model building

```
X = data.iloc[:,1:]
y = data.iloc[:,0]
RANDOM_STATE = 42
X_train, X_test, y_train, y_test = train_test_split(X,y,test_size = 0.25,random_state=RANDOM_STATE)
print(f"Train data shape of X = {X_train.shape} and Y = {y_train.shape}")
print(f"Test data shape of X = {X_test.shape} and Y = {y_test.shape}")
scaler = StandardScaler()
X_train = scaler.fit_transform(X_train)
X_test = scaler.transform(X_test)

#k-fold
fold = 3
```

GRADIENT BOOSTING

```
from sklearn.model_selection import GridSearchCV, train_test_split
from sklearn.ensemble import GradientBoostingClassifier
from sklearn.metrics import accuracy_score, confusion_matrix, classification_report
from sklearn.model_selection import cross_val_predict
from sklearn.model_selection import KFold

param_grid = {
    'n_estimators': [2000],
    'learning_rate': [0.001, 0.01, 0.1],
    'max_depth': [3, 5, 7],
    'criterion': ['friedman_mse', 'mse'],
    'loss': ['deviance', 'exponential']
}
grd_boost = GradientBoostingClassifier(random_state=RANDOM_STATE)
grid_search = GridSearchCV(estimator=grd_boost, param_grid=param_grid, cv=5, scoring='accuracy')
grid_search.fit(X_train, y_train)
print("Best Parameters:", grid_search.best_params_)
print("Best Score:", grid_search.best_score_)
best_grd_boost = grid_search.best_estimator_
best_grd_boost.fit(X_train, y_train)
y_pred_test_gb = best_grd_boost.predict(X_test)
conf_matrix = confusion_matrix(y_test, y_pred_test_gb)
conf_matrix = confusion_matrix(y_test, y_pred_test_gb)
accuracy_gb = accuracy_score(y_test, y_pred_test_gb)
report = classification_report(y_test, y_pred_test_gb)

print("Confusion Matrix (Test Data):\n", conf_matrix)
print("Accuracy:", accuracy_gb)
print("Classification Report (Test Data):\n", report)
kf_gb = KFold(n_splits=fold, shuffle=True, random_state=RANDOM_STATE)
y_pred_cv_gb = cross_val_predict(best_grd_boost, X_train, y_train, cv=kf_gb)
conf_matrix_cv = confusion_matrix(y_train, y_pred_cv_gb)
accuracy_cv_gb = accuracy_score(y_train, y_pred_cv_gb)
report_cv = classification_report(y_train, y_pred_cv_gb)

print("Confusion Matrix (Cross-Validation):\n", conf_matrix_cv)
print("Cross-Validation Accuracy:", accuracy_cv_gb)
print("Classification Report (Cross-Validation):\n", report_cv)
```

```
#Calculation of ROC Curve metrics and graph plotting
from sklearn.preprocessing import LabelBinarizer
from sklearn.metrics import roc_auc_score, roc_curve
import matplotlib.pyplot as plt

best_grd_boost.fit(X_train, y_train)
y_prob_gb = best_grd_boost.predict_proba(X_test)
label_binarizer = LabelBinarizer()
y_test_bin = label_binarizer.fit_transform(y_test)

fpr_gb, tpr_gb, thresholds_gb = roc_curve(y_test_bin, y_prob_gb[:,1],pos_label=1)

# roc curve for tpr = fpr
random_probs = [0 for i in range(len(y_test_bin))]
p_fpr, p_tpr, _ = roc_curve(y_test_bin, random_probs, pos_label=1)


# auc scores
roc_auc_gb = roc_auc_score(y_test_bin, y_prob_gb[:,1])
print(roc_auc_gb)


# matplotlib
plt.style.use('seaborn')

# plot roc curves
plt.plot(fpr_gb, tpr_gb, linestyle='--',color='orange', label=f'ROC Curve (AUC = {roc_auc_gb:.2f})')
plt.plot(p_fpr, p_tpr, linestyle='--', color='blue')
# title
plt.title('ROC curve')
# x label
plt.xlabel('False Positive Rate')
# y label
plt.ylabel('True Positive rate')
plt.legend(loc='best')
plt.savefig('ROC',dpi=300)
plt.show();
```

```
##FEATURE IMPORTANCE
X = data.drop('Sex', axis=1)
features=[]
for columns in X.columns:
    features.append(columns)
imp_features = best_grd_boost.feature_importances_
importances = best_grd_boost.feature_importances_

# Criar um DataFrame com as importâncias e nomes das features
feature_importance_gb = pd.DataFrame({'Feature': features, 'Importance': importances})

# Ordenar o DataFrame por importâncias em ordem decrescente
feature_importance_sorted = feature_importance_gb.sort_values('Importance', ascending=True)

# Criar um degradê de cores azuis
colors = plt.cm.Blues(np.linspace(0.2, 1, len(feature_importance_sorted)))

# Criar o gráfico de barras horizontais com o degradê de cores
plt.figure(figsize=(10, 6))
bars = plt.barh(feature_importance_sorted['Feature'], feature_importance_sorted['Importance'], color=colors)
plt.xlabel('Importance')
plt.ylabel('Feature')
plt.title('Feature Importance')

# Adicionar valor numérico nas barras
for bar in bars:
    plt.text(bar.get_width(), bar.get_y() + bar.get_height()/2, round(bar.get_width(), 4),
             va='center', ha='left', fontsize=10, color='white')

# Remover bordas do gráfico
plt.gca().spines['top'].set_visible(False)
plt.gca().spines['right'].set_visible(False)

# Adicionar uma grade leve
plt.grid(axis='x', linestyle='--', alpha=0.7)

# Tornar o fundo branco
plt.gca().set_facecolor('white')

# Ajustar o layout
plt.tight_layout()

plt.show()

plt.savefig('figimpKNN.jpg', dpi=300, bbox_inches='tight')
```

LOGISTIC REGRESSION

```
from sklearn.model_selection import GridSearchCV, train_test_split, cross_val_predict
from sklearn.linear_model import LogisticRegression
from sklearn.metrics import accuracy_score, confusion_matrix, classification_report
from sklearn.model_selection import KFold

param_grid = {

    'C': [0.001, 0.005, 0.01, 0.02, 0.03, 0.05, 0.1, 0.2, 0.3, 0.5, 1, 5, 10, 100],
    'penalty': ['l1', 'l2', 'elasticnet', 'none'],
    'max_iter':[50, 100, 300, 500, 1000],
    'solver': ['newton-cg', 'lbfgs', 'liblinear', 'sag', 'saga'],
    'l1_ratio': [0.2, 0.4, 0.6, 0.8]
}
log_reg = LogisticRegression(random_state=RANDOM_STATE)
grid_search = GridSearchCV(estimator=log_reg, param_grid=param_grid, cv=5, scoring='accuracy')
grid_search.fit(X_train, y_train)
print("Best Parameters:", grid_search.best_params_)
print("Best Score:", grid_search.best_score_)
best_log_reg = grid_search.best_estimator_
best_log_reg.fit(X_train, y_train)
y_pred_test_lr = best_log_reg.predict(X_test)
conf_matrix = confusion_matrix(y_test, y_pred_test_lr)
accuracy_lr = accuracy_score(y_test, y_pred_test_lr)
report = classification_report(y_test, y_pred_test_lr)

print("Confusion Matrix (Test Data):\n", conf_matrix)
print("Accuracy:", accuracy_lr)
print("Classification Report (Test Data):\n", report)
kf_lr = KFold(n_splits=fold, shuffle=True, random_state=RANDOM_STATE)
y_pred_cv_lr = cross_val_predict(best_log_reg, X_train, y_train, cv=kf_lr)
conf_matrix_cv = confusion_matrix(y_train, y_pred_cv_lr)
accuracy_cv_lr = accuracy_score(y_train, y_pred_cv_lr)
report_cv = classification_report(y_train, y_pred_cv_lr)

print("Confusion Matrix (Cross-Validation):\n", conf_matrix_cv)
print("Cross-Validation Accuracy:", accuracy_cv_lr)
print("Classification Report (Cross-Validation):\n", report_cv)


#Calculation of ROC Curve metrics and graph plotting
best_log_reg.fit(X_train, y_train)
y_prob_lr = best_log_reg.predict_proba(X_test)

fpr_lr, tpr_lr, thresholds_lr = roc_curve(y_test_bin, y_prob_lr[:,1],pos_label=1)

# auc scores
roc_auc_lr = roc_auc_score(y_test_bin, y_prob_lr[:,1])
print(roc_auc_lr)


# matplotlib
plt.style.use('seaborn')
```

```
# plot roc curves
plt.plot(fpr_lr, tpr_lr, linestyle='--',color='green', label=f'ROC Curve (AUC = {roc_auc_lr:.2f})')
plt.plot(p_fpr, p_tpr, linestyle='--', color='blue')
# title
plt.title('ROC curve')
# x label
plt.xlabel('False Positive Rate')
# y label
plt.ylabel('True Positive rate')
plt.legend(loc='best')
plt.savefig('ROC',dpi=300)
plt.show();
```

```
#FEATURE IMPORTANCE
model = best_log_reg
coefficients = model.coef_[0]
feature_importance_lr = pd.DataFrame({'Feature': X.columns, 'Importance': np.abs(coefficients)})
feature_importance_lr = feature_importance_lr.sort_values('Importance', ascending=True)
colors = plt.cm.Blues(np.linspace(0.2, 1, len(feature_importance_lr)))
plt.figure(figsize=(10, 6))
bars = plt.barh(feature_importance_lr['Feature'], feature_importance_lr['Importance'], color=colors)
plt.xlabel('Importance')
plt.ylabel('Feature')
plt.title('Feature Importance')
for bar in bars:
    plt.text(bar.get_width(), bar.get_y() + bar.get_height()/2, round(bar.get_width(), 4),
             va='center', ha='left', fontsize=10, color='white')
plt.gca().spines['top'].set_visible(False)
plt.gca().spines['right'].set_visible(False)
plt.grid(axis='x', linestyle='--', alpha=0.7)
plt.gca().set_facecolor('white')
plt.tight_layout()

plt.show()
```

SVM

```
from sklearn.model_selection import GridSearchCV, cross_val_predict
from sklearn.svm import SVC
from sklearn.metrics import accuracy_score, confusion_matrix, classification_report
import numpy as np

param_grid = {
    'C': [0.001, 0.1, 0.9, 1.5, 2.3,23, 50, 100],
    'kernel': ['linear','rbf'],
    'gamma': ['auto','scale']
}

svm = SVC(probability=True, random_state=RANDOM_STATE)
grid_search = GridSearchCV(estimator=svm, param_grid=param_grid, cv=5, scoring='accuracy')
grid_search.fit(X_train, y_train)
print("Best Parameters:", grid_search.best_params_)
print("Best Score:", grid_search.best_score_)
best_svm = grid_search.best_estimator_
best_svm.fit(X_train, y_train)
y_pred_test_svm = best_svm.predict(X_test)
conf_matrix = confusion_matrix(y_test, y_pred_test_svm)
accuracy_svm = accuracy_score(y_test, y_pred_test_svm)
report = classification_report(y_test, y_pred_test_svm)

print("Confusion Matrix (Test Data):\n", conf_matrix)
print("Accuracy:", accuracy_svm)
print("Classification Report (Test Data):\n", report)

kf_svm = KFold(n_splits=fold, shuffle=True, random_state=RANDOM_STATE)
y_pred_cv_svm = cross_val_predict(best_svm, X_train, y_train, cv=kf_svm)
conf_matrix_cv = confusion_matrix(y_train, y_pred_cv_svm)
accuracy_cv_svm = accuracy_score(y_train, y_pred_cv_svm)
report_cv = classification_report(y_train, y_pred_cv_svm)

print("Confusion Matrix (Cross-Validation):\n", conf_matrix_cv)
print("Cross-Validation Accuracy:", accuracy_cv_svm)
print("Classification Report (Cross-Validation):\n", report_cv)
```

```
#Calculation of ROC Curve metrics and graph plotting
best_svm.fit(X_train, y_train)
y_prob_svm = best_svm.predict_proba(X_test)

fpr_svm, tpr_svm, thresholds_svm = roc_curve(y_test_bin, y_prob_svm[:,1],pos_label=1)

# auc scores
roc_auc_svm = roc_auc_score(y_test_bin, y_prob_svm[:,1])
print(roc_auc_svm)


# matplotlib
plt.style.use('seaborn')

# plot roc curves
plt.plot(fpr_svm, tpr_svm, linestyle='--',color='red', label=f'ROC Curve (AUC = {roc_auc_svm:.2f})')
plt.plot(p_fpr, p_tpr, linestyle='--', color='blue')
# title
plt.title('ROC curve')
# x label
plt.xlabel('False Positive Rate')
# y label
plt.ylabel('True Positive rate')
plt.legend(loc='best')
```

```
plt.legend(loc='best')
plt.savefig('ROC',dpi=300)
plt.show();
```

KNN

```
from sklearn.model_selection import GridSearchCV, cross_val_predict
from sklearn.neighbors import KNeighborsClassifier
from sklearn.metrics import accuracy_score, confusion_matrix, classification_report

param_grid = {
   'n_neighbors': [1, 3, 5, 7, 10, 15, 100, 1000],
    'weights': ['uniform', 'distance'],
    'p': [0.001, 0.1, 1, 3, 5, 7, 10, 15, 100, 1000],
    'leaf_size': [0.001, 0.1, 1, 3, 5, 7, 10, 15, 100, 1000]
}

knn = KNeighborsClassifier()
grid_search = GridSearchCV(estimator=knn, param_grid=param_grid, cv=5, scoring='accuracy')
grid_search.fit(X_train, y_train)
print("Best Parameters:", grid_search.best_params_)
print("Best Score:", grid_search.best_score_)
best_knn = grid_search.best_estimator_
best_knn.fit(X_train, y_train)
y_pred_test_knn = best_knn.predict(X_test)
conf_matrix = confusion_matrix(y_test, y_pred_test_knn)
accuracy_knn = accuracy_score(y_test, y_pred_test_knn)
report = classification_report(y_test, y_pred_test_knn)

print("Confusion Matrix (Test Data):\n", conf_matrix)
print("Accuracy:", accuracy_knn)
print("Classification Report (Test Data):\n", report)
kf_knn = KFold(n_splits=fold, shuffle=True, random_state=RANDOM_STATE)
y_pred_cv_knn = cross_val_predict(best_knn, X_train, y_train, cv=kf_knn)

conf_matrix_cv = confusion_matrix(y_train, y_pred_cv_knn)
accuracy_cv_knn = accuracy_score(y_train, y_pred_cv_knn)
report_cv = classification_report(y_train, y_pred_cv_knn)

print("Confusion Matrix (Cross-Validation):\n", conf_matrix_cv)
print("Cross-Validation Accuracy:", accuracy_cv_knn)
print("Classification Report (Cross-Validation):\n", report_cv)
```

```
#Calculation of ROC Curve metrics and graph plotting
best_knn.fit(X_train, y_train)
y_prob_knn = best_knn.predict_proba(X_test)

fpr_knn, tpr_knn, thresholds_knn = roc_curve(y_test_bin, y_prob_knn[:,1],pos_label=1)

# auc scores
roc_auc_knn = roc_auc_score(y_test_bin, y_prob_knn[:,1])
print(roc_auc_knn)


# matplotlib
plt.style.use('seaborn')

# plot roc curves
plt.plot(fpr_knn, tpr_knn, linestyle='--',color='deeppink', label=f'ROC Curve (AUC = {roc_auc_knn:.2f})')
plt.plot(p_fpr, p_tpr, linestyle='--', color='blue')
# title
plt.title('ROC curve')
# x label
plt.xlabel('False Positive Rate')
# y label
plt.ylabel('True Positive rate')
plt.legend(loc='best')
plt.savefig('ROC',dpi=300)
plt.show();
```

MLP CLASSIFIER

```
from sklearn.neural_network import MLPClassifier
from sklearn.model_selection import GridSearchCV, cross_val_predict
from sklearn.metrics import accuracy_score, confusion_matrix, classification_report

param_grid = {
    'hidden_layer_sizes': [10, 100, 1000],
    'alpha': [ 0.01, 0.1, 1.0],
    'learning_rate_init': [0.01, 0.1, 1],
    'activation': ['relu', 'logistic', 'tanh'],
    'max_iter': [50, 100, 1000],
    'solver': ['lbfgs', 'sgd', 'adam']
}

mlp_clf = MLPClassifier(random_state=42)
grid_search = GridSearchCV(estimator=mlp_clf, param_grid=param_grid, cv=5, scoring='accuracy')

grid_search.fit(X_train, y_train)

print("Melhores Parâmetros Encontrados:")
print(grid_search.best_params_)
best_mlp_clf = grid_search.best_estimator_
y_pred_mlp_test = best_mlp_clf.predict(X_test)
conf_matrix_mlp_test = confusion_matrix(y_test, y_pred_mlp_test)
accuracy_mlp_test = accuracy_score(y_test, y_pred_mlp_test)
```

```
report_mlp_test = classification_report(y_test, y_pred_mlp_test)

print("Confusion Matrix (Test Data):\n", conf_matrix_mlp_test)
print("Accuracy (Test Data):", accuracy_mlp_test)
print("Classification Report (Test Data):\n", report_mlp_test)
kf_mlp = KFold(n_splits=fold, shuffle=True, random_state=RANDOM_STATE)
y_pred_mlp_cv = cross_val_predict(best_mlp_clf, X_train, y_train, cv=kf_mlp)
conf_matrix_mlp_cv = confusion_matrix(y_train, y_pred_mlp_cv)
accuracy_mlp_cv = accuracy_score(y_train, y_pred_mlp_cv)
report_mlp_cv = classification_report(y_train, y_pred_mlp_cv)

print("Confusion Matrix (Cross-Validation):\n", conf_matrix_mlp_cv)
print("Cross-Validation Accuracy:", accuracy_mlp_cv)
print("Classification Report (Cross-Validation):\n", report_mlp_cv)



#Calculation of ROC Curve metrics and graph plotting
best_mlp_clf.fit(X_train, y_train)
y_prob_mlp = best_mlp_clf.predict_proba(X_test)

fpr_mlp, tpr_mlp, thresholds_mlp = roc_curve(y_test_bin, y_prob_mlp[:,1],pos_label=1)

# auc scores
roc_auc_mlp = roc_auc_score(y_test_bin, y_prob_mlp[:,1])
print(roc_auc_mlp)


# matplotlib
plt.style.use('seaborn')

# plot roc curves
plt.plot(fpr_mlp, tpr_mlp, linestyle='--',color='indigo', label=f'ROC Curve (AUC = {roc_auc_mlp:.2f})')
plt.plot(p_fpr, p_tpr, linestyle='--', color='blue')
# title
plt.title('ROC curve')
# x label
plt.xlabel('False Positive Rate')
# y label
plt.ylabel('True Positive rate')
plt.legend(loc='best')
plt.savefig('ROC',dpi=300)
plt.show();
```

DECISION TREE

```
from sklearn.model_selection import GridSearchCV, train_test_split, cross_val_predict
from sklearn.tree import DecisionTreeClassifier
from sklearn.metrics import accuracy_score, confusion_matrix, classification_report

param_grid = {
    'criterion': ['gini', 'entropy'],
    'splitter': ['best', 'random'],
    'max_depth': [None, 5, 10, 15],
}

tree_clf = DecisionTreeClassifier(random_state=RANDOM_STATE)
grid_search = GridSearchCV(estimator=tree_clf, param_grid=param_grid, cv=5, scoring='accuracy')
grid_search.fit(X_train, y_train)
print("Best Parameters:", grid_search.best_params_)
print("Best Score:", grid_search.best_score_)
best_tree_clf = grid_search.best_estimator_
best_tree_clf.fit(X_train, y_train)
y_pred_test_dt = best_tree_clf.predict(X_test)
conf_matrix = confusion_matrix(y_test, y_pred_test_dt)
accuracy_dt = accuracy_score(y_test, y_pred_test_dt)
report = classification_report(y_test, y_pred_test_dt)

print("Confusion Matrix (Test Data):\n", conf_matrix)
print("Accuracy:", accuracy_dt)
print("Classification Report (Test Data):\n", report)
kf_dt = KFold(n_splits=fold, shuffle=True, random_state=RANDOM_STATE)
y_pred_cv_dt = cross_val_predict(best_tree_clf, X_train, y_train, cv=kf_dt)
conf_matrix_cv = confusion_matrix(y_train, y_pred_cv_dt)
accuracy_cv_dt = accuracy_score(y_train, y_pred_cv_dt)
report_cv = classification_report(y_train, y_pred_cv_dt)

print("Confusion Matrix (Cross-Validation):\n", conf_matrix_cv)
print("Cross-Validation Accuracy:", accuracy_cv_dt)
print("Classification Report (Cross-Validation):\n", report_cv)



#Calculation of ROC Curve metrics and graph plotting
best_tree_clf.fit(X_train, y_train)
y_prob_tree = best_tree_clf.predict_proba(X_test)

fpr_tree, tpr_tree, thresholds_tree = roc_curve(y_test_bin, y_prob_tree[:,1],pos_label=1)

# auc scores
roc_auc_tree = roc_auc_score(y_test_bin, y_prob_tree[:,1])
print(roc_auc_tree)


# matplotlib
plt.style.use('seaborn')

# plot roc curves
plt.plot(fpr_tree, tpr_tree, linestyle='--',color='steelblue', label=f'ROC Curve (AUC = {roc_auc_tree:.2f})')
plt.plot(p_fpr, p_tpr, linestyle='--', color='blue')
# title
plt.title('ROC curve')
```

```
# x label
plt.xlabel('False Positive Rate')
# y label
plt.ylabel('True Positive rate')
plt.legend(loc='best')
plt.savefig('ROC',dpi=300)
plt.show();


#FEATURE IMPORTANCE
X_2 = []
X_2
features_2 =[]
imp_features_dt2 = []
df_imp_features_dt2 = []
X_2 = data.drop('Sex', axis=1)
features_2 = []

for column in X_2.columns:
    features_2.append(column)

imp_features_dt2 = best_tree_clf.feature_importances_
df_imp_features_dt2 = pd.DataFrame({"features": features_2, "weights": imp_features_dt2})
df_imp_features_dt2_sorted = df_imp_features_dt2.sort_values(by='weights', ascending=True)
plt.figure(figsize=(10, 6))
colors = plt.cm.Blues(np.linspace(0.2, 1, len(df_imp_features_dt2_sorted)))
bars = plt.barh(df_imp_features_dt2_sorted['features'], df_imp_features_dt2_sorted['weights'], color=colors)
plt.xlabel('Importance')
plt.ylabel('Feature')
plt.title('Feature Importance')
for bar in bars:
    plt.text(bar.get_width(), bar.get_y() + bar.get_height()/2, round(bar.get_width(), 4),
             va='center', ha='left', fontsize=10, color='white')
plt.gca().spines['top'].set_visible(False)
plt.gca().spines['right'].set_visible(False)
plt.grid(axis='x', linestyle='--', alpha=0.7)
plt.gca().set_facecolor('white')
plt.tight_layout()

plt.show()
```

RANDOM FOREST

```
from sklearn.ensemble import RandomForestClassifier
from sklearn.model_selection import GridSearchCV, train_test_split, cross_val_predict
from sklearn.metrics import accuracy_score, confusion_matrix, classification_report

# Dividir os dados com random_state definido
RANDOM_STATE = 42
X_train, X_test, y_train, y_test = train_test_split(X, y, test_size=0.25, random_state=RANDOM_STATE)

# Definir os hiperparâmetros e seus possíveis valores para o Grid Search
param_grid = {
    'n_estimators': [5, 50, 200],
    'max_depth': [0, 10, 20],
    'min_samples_split': [2, 10, 15],
    'min_samples_leaf': [1, 4, 6],
    'max_features': ['auto', 'sqrt'],
    'criterion': ['gini', 'entropy']
}

# Criar o modelo Random Forest Classifier
forest_clf = RandomForestClassifier(random_state=RANDOM_STATE)

# Criar o objeto GridSearchCV com o random_state definido
grid_search = GridSearchCV(estimator=forest_clf, param_grid=param_grid, cv=5, scoring='accuracy')

# Realizar o Grid Search junto com a validação cruzada
grid_search.fit(X_train, y_train)

# Imprimir os melhores parâmetros encontrados pelo Grid Search
print("Best Parameters:", grid_search.best_params_)

# Imprimir a melhor pontuação do Grid Search
print("Best Score:", grid_search.best_score_)

# Treinar o modelo final com todos os dados de treinamento usando os melhores parâmetros
best_forest_clf = grid_search.best_estimator_
best_forest_clf.fit(X_train, y_train)

# Fazer previsões no conjunto de teste usando o modelo com os melhores parâmetros
y_pred_test_rf = best_forest_clf.predict(X_test)

# Calcular a matriz de confusão
conf_matrix = confusion_matrix(y_test, y_pred_test_rf)

# Calcular e imprimir as métricas no conjunto de teste
accuracy_rf = accuracy_score(y_test, y_pred_test_rf)
report = classification_report(y_test, y_pred_test_rf)

print("Confusion Matrix (Test Data):\n", conf_matrix)
print("Accuracy:", accuracy_rf)
print("Classification Report (Test Data):\n", report)

# Realizar previsões com validação cruzada
kf_rf = KFold(n_splits=fold, shuffle=True, random_state=RANDOM_STATE)
y_pred_cv_rf = cross_val_predict(best_forest_clf, X_train, y_train, cv=kf_rf)

# Calcular e imprimir as métricas da validação cruzada
conf_matrix_cv = confusion_matrix(y_train, y_pred_cv_rf)
```

```
accuracy_cv_rf = accuracy_score(y_train, y_pred_cv_rf)
report_cv = classification_report(y_train, y_pred_cv_rf)

print("Confusion Matrix (Cross-Validation):\n", conf_matrix_cv)
print("Cross-Validation Accuracy:", accuracy_cv_rf)
print("Classification Report (Cross-Validation):\n", report_cv)



#Calculation of ROC Curve metrics and graph plotting
best_forest_clf.fit(X_train, y_train)
y_prob_forest = best_forest_clf.predict_proba(X_test)

fpr_forest, tpr_forest, thresholds_forest = roc_curve(y_test_bin, y_prob_forest[:,1],pos_label=1)

# auc scores
roc_auc_forest = roc_auc_score(y_test_bin, y_prob_forest[:,1])
print(roc_auc_forest)


# matplotlib
plt.style.use('seaborn')

# plot roc curves
plt.plot(fpr_forest, tpr_forest, linestyle='--',color='maroon', label=f'ROC Curve (AUC = {roc_auc_forest:.2f})')
plt.plot(p_fpr, p_tpr, linestyle='--', color='blue')
# title
plt.title('ROC curve')
# x label
plt.xlabel('False Positive Rate')
# y label
plt.ylabel('True Positive rate')
plt.legend(loc='best')
plt.savefig('ROC',dpi=300)
plt.show();


#FEATURE IMPORTANCE

X_3 = data.drop('Sex', axis=1)
features_3 = []

for column in X_3.columns:
    features_3.append(column)

imp_features_dt3 = best_forest_clf.feature_importances_
df_imp_features_dt3 = pd.DataFrame({"features": features_3, "weights": imp_features_dt3})
df_imp_features_dt3_sorted = df_imp_features_dt3.sort_values(by='weights', ascending=True)
plt.figure(figsize=(10, 6))
colors = plt.cm.Blues(np.linspace(0.2, 1, len(df_imp_features_dt3_sorted)))
bars = plt.barh(df_imp_features_dt3_sorted['features'], df_imp_features_dt3_sorted['weights'], color=colors)
plt.xlabel('Importance')
plt.ylabel('Feature')
plt.title('Feature Importance')
for bar in bars:
    plt.text(bar.get_width(), bar.get_y() + bar.get_height()/2, round(bar.get_width(), 4),
             va='center', ha='left', fontsize=10, color='white')
plt.gca().spines['top'].set_visible(False)
plt.gca().spines['right'].set_visible(False)
plt.grid(axis='x', linestyle='--', alpha=0.7)
plt.gca().set_facecolor('white')
plt.tight_layout()

plt.show()
```

plot - ROC

```
# Plotar a curva ROC para os modelos Gradient Boosting Classifier e Regressão Logística
plt.figure(figsize=(10, 8))  # Definir o tamanho da figura

plt.plot(fpr_lr, tpr_lr, color='red', lw=2, linestyle='--', label='LR ROC curve (area = %0.2f)' % roc_auc_lr)
plt.plot(fpr_svm, tpr_svm, color='green', lw=2,linestyle='--', label='SVM ROC curve (area = %0.2f)' % roc_auc_svm)
plt.plot(fpr_knn, tpr_knn, color='blue', lw=2,linestyle='--', label='KNN ROC curve (area = %0.2f)' % roc_auc_knn)
plt.plot(fpr_forest, tpr_forest, color='deeppink',linestyle='--', lw=2, label='RF ROC curve (area = %0.2f)' % roc_auc_forest)
plt.plot(fpr_gb, tpr_gb, color='orange', lw=2,linestyle='--', label='GB ROC curve (area = %0.2f)' % roc_auc_gb)
plt.plot(fpr_mlp, tpr_mlp, color='purple', lw=2,linestyle='--', label='MLP ROC curve (area = %0.2f)' % roc_auc_mlp)
plt.plot(fpr_tree, tpr_tree, color='gray', lw=2,linestyle='--', label='DT ROC curve (area = %0.2f)' % roc_auc_tree)

plt.plot([0, 1], [0, 1], color='navy', lw=2, linestyle='-')
plt.xlim([0.0, 1.0])
plt.ylim([0.0, 1.05])
plt.xlabel('False Positive Rate (1- Specificity)')
plt.ylabel('True Positive Rate (Sensitivity)')
plt.title('Receiver Operating Characteristic')
plt.legend(loc="lower right")
plt.grid(True)  # Adicionar linhas de grade

# Salvar a figura em alta resolução
plt.savefig('roc_curve.jpg', dpi=300, bbox_inches='tight')

plt.show()
```
